# Proteomics and network pharmacology of Ganshu Nuodan capsules in the prevention of alcoholic liver disease

**DOI:** 10.3389/fendo.2023.1229777

**Published:** 2023-09-18

**Authors:** Xiaonan Yang, Lei Wang, Xuejie Cui, Jing Zhang, Ying Liang, Zhaojing Luo, Bingxue Zhou, Zheng Jiang, Rachel Y. H. Yang, Yi Wu, Kunhua Wei, Maobo Du, Shuangshuang Qin, Chen Dai, Guoliang Zhao

**Affiliations:** ^1^ Guangxi Key Laboratory of Medicinal Resources Protection and Genetic Improvement, National Engineering Research Center for Southwest Endangered Medicinal Resources Development, National Center for TCM Inheritance and Innovation, Guangxi Botanical Garden of Medicinal Plants, Nanning, China; ^2^ Department of Ultrasound, Shandong Provincial Hospital Affiliated To Shandong First Medical University, Jinan, China; ^3^ Ministry of Education (MOE) Joint International Research Laboratory of Animal Health and Food Safety, College of Veterinary Medicine, Nanjing Agricultural University, Nanjing, China; ^4^ College of Pharmacy, Shandong University of Traditional Chinese Medicine, Jinan, China; ^5^ Department of Traditional Chinese Medicine, Shandong Mental Health Center, Shandong University, Jinan, Shandong, China; ^6^ Upper School, La Jolla Country Day School, La Jolla, CA, United States; ^7^ Institute of Chinese Materia Medica, China Academy of Chinese Medical Sciences, Beijing, China; ^8^ College of Life Sciences, Nanjing Agricultural University, Nanjing, China; ^9^ Department of Gastroenterology, The First Affiliated Hospital of Shandong First Medical University & Shandong Provincial Qianfoshan Hospital, Jinan, China

**Keywords:** alcohol liver disease, component identification, molecular docking, network pharmacology, Proteomics

## Abstract

**Introduction:**

Ganshu Nuodan is a liver-protecting dietary supplement composed of *Ganoderma lucidum* (*G. lucidum*) spore powder, *Pueraria montana* (Lour.) Merr. (*P. montana*), *Salvia miltiorrhiza* Bunge (*S. miltiorrhiza*) and *Astragalus membranaceus* (Fisch.) Bunge. (*A. membranaceus*). However, its pharmacodynamic material basis and mechanism of action remain unknown.

**Methods:**

A mouse model of acute alcohol liver disease (ALD) induced by intragastric administration of 50% alcohol was used to evaluate the hepatoprotective effect of Ganshu Nuodan. The chemical constituents of Ganshu Nuodan were comprehensively identified by UPLC-QTOF/MS, and then its pharmacodynamic material basis and potential mechanism of action were explored by proteomics and network pharmacology.

**Results:**

Ganshu Nuodan could ameliorate acute ALD, which is mainly manifested in the significant reduction of alanine aminotransferase (ALT) and aspartate aminotransferase (AST) in serum and malondialdehyde (MDA) content in liver and the remarkably increase of glutathione (GSH) content and superoxide dismutase (SOD) activity in liver. Totally 76 chemical constituents were identified from Ganshu Nuodan by UPLC-QTOF/MS, including 21 quinones, 18 flavonoids, 11 organic acids, 7 terpenoids, 5 ketones, 4 sterols, 3 coumarins and 7 others. Three key signaling pathways were identified via proteomics studies, namely Arachidonic acid metabolism, Retinol metabolism, and HIF-1 signaling pathway respectively. Combined with network pharmacology and molecular docking, six key targets were subsequently obtained, including Ephx2, Lta4h, Map2k1, Stat3, Mtor and Dgat1. Finally, these six key targets and their related components were verified by molecular docking, which could explain the material basis of the hepatoprotective effect of Ganshu Nuodan.

**Conclusion:**

Ganshu Nuodan can protect acute alcohol-induced liver injury in mice by inhibiting oxidative stress, lipid accumulation and apoptosis. Our study provides a scientific basis for the hepatoprotective effect of Ganshu Nuodan in acute ALD mice and supports its traditional application.

## 1 Introduction

According to the World Health Organization’s latest Global Status Report on Alcohol, the total alcohol per capita consumption increased from 5.5 L in 2005 to 6.4 L in 2016. One of the main reasons is the prevalence of alcohol drinking culture. Heavy drinking causes a range of ALDs, including simple steatosis, steatohepatitis, cirrhosis, and end-stage hepatocellular carcinoma, among which the mortality rate of acute alcoholic hepatitis can be as high as 50%. With the progress of society, people’s health awareness has gradually improved, and more and more liver protection drugs have emerged.

TCM has a long history of prevention and treatment of liver disease based on the relevant information recorded in Shennong Herbal Classic and Treatise on Febrile. Due to its advantages of stable supply, long-term efficacy and small side effects, it has attracted more attention from the public in recent years. Ganshu Nuodan consists of four herbs, including *G. lucidum* spore powder, *P. montana*, *S. miltiorrhiza* and *A. membranaceus*. *A. membranaceus* is considered to benefit blood circulation and promote pus discharge and new tissue growth. The other three herbs mainly exert liver protection through antioxidation. Among them, *G. lucidum* spore powder plays a major role thanks to its rich triterpenoids and organic acids. It is reported that *P. montana* can be used to treat post-alcoholic coma vomiting, and therefore it is often used as an anti-drinking agent. Combination of *A. membranaceus* and *P. montana* has been used as a clinically effective formula for treating liver damage diseases for many years in China.

However, since TCM has the characteristics of multi-component, multi-target and multi-link comprehensive action, the chemical components and mechanism have become two major challenges in the research of TCM.

With the emergence of liquid chromatography-mass spectrometry technology, the comprehensive characterization of TCM components has been realized. For example, Baofu Lin characterized 107 chemical components in the Chinese herbal formula Li Chang Decoction by UPLC-QTOF/MS (1). Through UPLC-LTQ-Orbitrap-MSn technology, 80 chemical components were characterized from Bu Shen Yi Sui Capsule (2). On the other hand, with the gradual completion of genome sequencing of various model organisms, proteomics has emerged in the mechanism studies of TCM. Elva Morretta studied the mechanism of pharmaceutical-grade Triticum vulgare extract in speeding up keratinocyte healing by label-free quantitative proteomics and found that many of the up-regulated proteins were involved in promoting wound-healing-related processes, such as modulating cell-cell interaction and communication, cell proliferation and differentiation, and enhancing cell adhesion and migration (3). Hareram Birla investigated the neuroprotective molecular mechanism of Tinospora cordifolia in a rotenone-intoxicated mouse model using a proteomics approach. Network pharmacology is a fusion of systems biology, network biology, computational biology, multi-directional pharmacology, molecular pharmacology, molecular dynamics and other multidisciplinary technologies and contents (4) and the construction and integration of “formulas-targets-pathways-disease” Hierarchical network (5). It analyzes the interaction between drugs and specific nodes in the network, and explores a new discipline of drug-body interaction from a systematic and holistic perspective (6). This discipline provides strong technical support for the research on the multi-target, multi-component, holistic and systemic mechanism of action of TCM, and helps to reveal the scientific connotation of TCM and discover drug targets.

In this experiment, UPLC-QTOF/MS combined with UNIFI software was used to comprehensively characterize the composition of Ganshu Nuodan. Then, the hepatoprotective effect of Ganshu Nuodan on ALD mice was evaluated, and related differential proteins were analyzed by label-free quantitative proteomics. Finally, combined with network pharmacology the multi-target mechanism of Ganshu Nuodan is described.

## 2 Materials and methods

### 2.1 Materials and reagents

Ganshu Nuodan was provided by Shanghai Aonokang Biotechnology Co., Ltd. (Shanghai, China). Bifendate (Beijing Xiehe Pharmaceutical Co., Ltd, Beijing, China) was used as a positive control for hepatoprotection, and ethanol absolute (Guangdong Guanghua Sci-Tech Co., Ltd, Guangdong, China) was used to induce acute ALD model. Kits for assessing the levels of ALT, AST, MDA, GSH and SOD were purchased from Nanjing Jiancheng Technology Co., Ltd. (Nanjing, China).

### 2.2 Sample preparation

Add 1g Ganshu Nuodan to 20 mL ultrapure water, sonicate for 30 min, the supernatant solution was transferred to a sample vial after passing through a 0.22 μm filter.

### 2.3 Animals

Male C57BL/6J mice (6-8 weeks old, weighing between 18 and 22 g) were supplied from the Center for Comparative Medicine of Yangzhou University (Jiangsu, China). The experimental animals were kept in the animal laboratory of Nanjing Agricultural University at room temperature of 22°C ± 2°C. The experiments were approved by the ethics committee of Nanjing Agricultural University, and the animal welfare and experimental procedures were incompliance with the regulations of Nanjing Agricultural University for the care and use of laboratory animals (Nanjing, China).

### 2.4 Anti-acute ALD study of Ganshu Nuodan

After 1 week of adaptive feeding, 48 C57BL/6J mice were randomly divided into 6 groups (n=8): control group, model group, positive control group, low-dose (1 g/kg Ganshu Nuodan) group, middle-dose (2 g/kg Ganshu Nuodan) group and high-dose (4 g/kg Ganshu Nuodan) group. After the drugs were intragastrically administrated for 15 days continuously, all the mice except the control group mice were given 50% alcohol to induce acute ALD model by an intragastric route at the volumn of 14 mL/kg. After that, the mice were anesthetized to collect blood and then sacrificed for liver collection. The serum was collected by centrifugation at 3500 rpm for 15 min for biochemical study. The liver tissues of mice were stripped, weighed, and partly fixed in 4% paraformaldehyde for histopathological examination. The remaining liver tissues were stored at -80°C for biochemical study and proteomics.

### 2.5 Compound identification of Ganshu Nuodan by UPLC-QTOF-MS analysis

The compound of Ganshu Nuodan were analyzed using a Waters ACQUITY UPLCTM liquid chromatograph and mass spectrometer (Waters Xevo G2-XS QTOF mass spectrometer) equipped with electrospray ionization (ESI) in negative and positive ion scanning mode.

For the chromatographic separation, an UPLC column (BEH C18 2.1 mm× 100 mm, 1.8 μm) was used with a flow rate, injection volume, and oven temperature of 0.3 mL/min, 5 μL, and 40°C, respectively. Water with 0.1% FA “A” and acetonitrile “B” were applied as the mobile phase using the gradient elution as follows: 0~1 min, 2% B, 1~ 22 min, 2%- 95% B; 22~ 26.4 min, 95% B; 26.4~ 26.5 min, 95%- 2% B; 26.5~ 30 min, 2% B. For the mass spectrometry, the desolvation gas flow rate and desolvation temperature were 600 L/h and 400°C, respectively. The scan range was from m/z 50 to 1000, capillary voltage 3 kV, cone voltage 30 V, MSE collision energy low energy set to 6 V, the high energy setting was 20- 45 V. The compounds of Ganshu Nuodan were identified through the comparison of their molecular formula, structural formula and MS/MS fragments with the self-built database (7), using UNIFI 2.0 software.

### 2.6 Proteomics

200 μg protein was added to a final concentration of 10 mM DTT 35 °C for 1.5 h, then restored to room temperature. IAM with final concentration of 20 mM was added at room temperature for 40 min. The sample was diluted 4-fold with 25 mM ammonium bicarbonate (pH=8). Trypsin was added at 50: 1 ratio of protein to trypsin and the sample solution was incubated at 37 °C overnight. C18 column was prepared in the order of washing with 200 μL 60% ACN, centrifuging at 5000 RCF for 2 min, 200 μL 0.2% FA washing once and centrifuging at 5000 RCF for 2 min. After the sample was loaded, the column was centrifuged at 5000 RCF for 2 min, followed by column washing with 200 μL 0.2% FA, centrifugation at RCF for 2 min, and compound elution with 60% ACN. The collected eluent was then lyophilized.

Mobile phase A (100% mass spectrometry grade water with 0.1% FA) and B (80% ACN, with 0.1% FA) were prepared. Tryptic peptides were reconstituted in MP A solution and injected for LC-MS/MS analysis. Chromatographic separation was carried out by the analytical column (Acclaim PepMap^®^ RSLC, C18, 75μm × 15cm, 3 μm, 100Å, Thermo Scientific) using a linear gradient of 3-35% buffer B at a flow rate of 0.3 μL/min over 70 min. The compensation voltage (CV) of FAIMS was -45V and -65V. The mass spectrometer was operated in data dependent analysis (DDA) mode with dynamic exclusion of 30 s and full-scan MS spectra (m/z 350–1500) with resolution of 60,000 (m/z 200), followed by fragmentation of the most intense ions within 1 s cycle time with high energy collisional dissociation (HCD), normalized collision energy (NCE) of 30.0 and resolution of 15,000 (m/z 200) in MS/MS scans. The obtained raw data was imported to Proteome Discoverer (Version. 2.4, Thermo Scientific) for protein identification.

### 2.7 Network pharmacology

The compounds identified from Ganshu Nuodan were imported into databases such as “Drug Bank” and “Swiss Target Prediction”, and the targets were obtained by taking Probability>0 as the screening condition. Then, the common targets and the significantly differentially expressed proteins obtained by proteomics were imported into Cytoscape 3.8.2. together with the pathways obtained by KEGG pathway enrichment analysis, and subsequently visualized by the “compounds-targets-pathways” network.

Molecular docking was used to ascertain the interaction between targets and their related compounds to obtain the crucial compound. Hence, key targets and related compounds were chosen for verification of molecular docking. Autodock software was used for compound-target molecular docking. The crystal structures of the targets were selected from the PDB website (http://www.rcsb.org/).

### 2.8 Statistcal analysis

Analysis was performed using Prism 8.0.1 software (Graphpad Software Inc., La Jolla, CA, USA). Data are presented as means ± standard error of the mean. Comparisons between groups were done using one-way analysis of variance followed by a Dunnett *post hoc* test. *P* < 0.05 was considered to be significantly different.

## 3 Results and discussion

### 3.1 Ganshu Nuodan shows a good liver-protected effect against acute ALD

**Figure 1:**
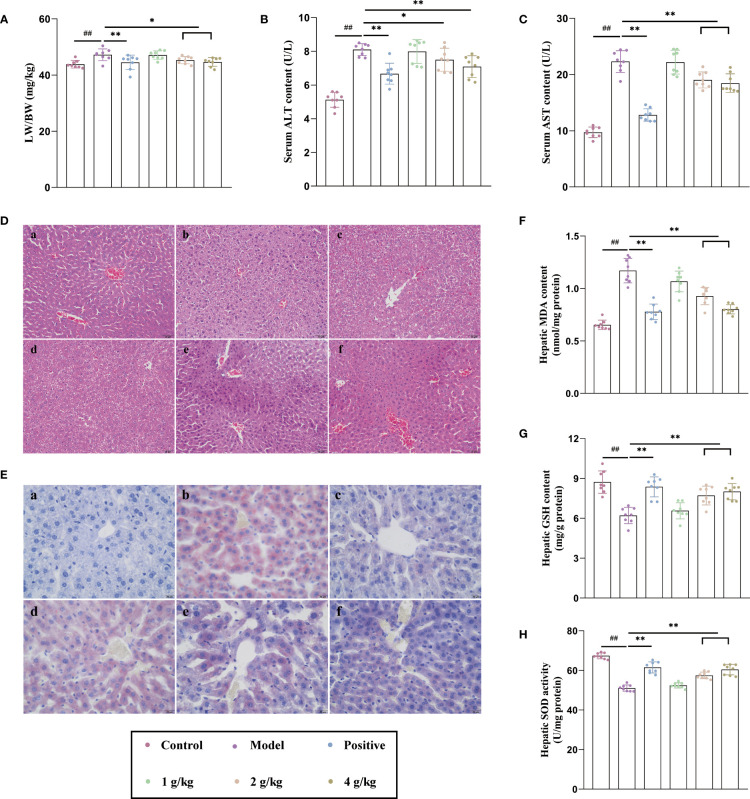
Ganshu Nuodan alleviates lipid accumulation, oxidative stress and degree of damage in the liver of alcohol induced mice. **(A)** The ratio of liver weight to body weight (LW/BW). **(B, C)** Serum ALT and AST contents. **(D, E)** Representative images of H&E (200×) and Oil-red O (400×) staining 533 of liver sections: (a) Control group, (b) Model group, (c) Positive control group, (d) 1 g/kg group, (e) 2 g/kg group, (f) 4 g/kg group. **(F, G)** Hepatic MDA and GSH contents. **(H)** Hepatic SOD activities.Compared with the normal group, ##p < 0.01; compared with the model group, *p < 0.05, **p < 0.01.

Alcohol remarkably induced liver morphological changes and increased the liver relative weight of mice, while Ganshu Nuodan significantly reversed the effects (**Figure 1A**). Ganshu Nuodan reduced the abnormally high levels of ALT (**Figure 1B**) and AST (**Figure 1C**), two important biochemical parameters reflecting liver function, in ALD mice, showing that Ganshu Nuodan had the function of liver-protection against ALD. As shown in **Figure 1D**, the hepatic cord of the control group was distributed with radical, while the hepatic cord of the model group was distributed disorderly, with vacuoles of different sizes in the cytoplasm. After Ganshu Nuodan treatment, the extent of liver injury was significantly ameliorated compared with model group, almost close to those of the control group.

The intake of alcohol could affect the normal metabolism of lipids, so that it accumulates *in vivo*, which could be seen from the results of oil red O staining (**Figure 1E**). Compared with the normal group, the model group showed obvious lipid aggregation while the administration group significantly improved this phenomenon in a dose-dependent manner. Alcohol could produce a large number of free radicals in the process of oxidative metabolism. The gradual accumulation and diffusion of free radicals will cause oxidative stress and destroy the structure and function of cells. This caused the increase of MDA content (**Figure 1F**), and the decrease of GSH content (**Figure 1G**) and SOD activity (**Figure 1H**), but Ganshu Nuodan reversed this phenomenon. These results indicated that Ganshu Nuodan alleviates lipid accumulation, oxidative stress and degree of damage in the livers of alcohol-induced mice.

### 3.2 Identification of compounds by UPLC-QTOF/MS

**Figure 2:**
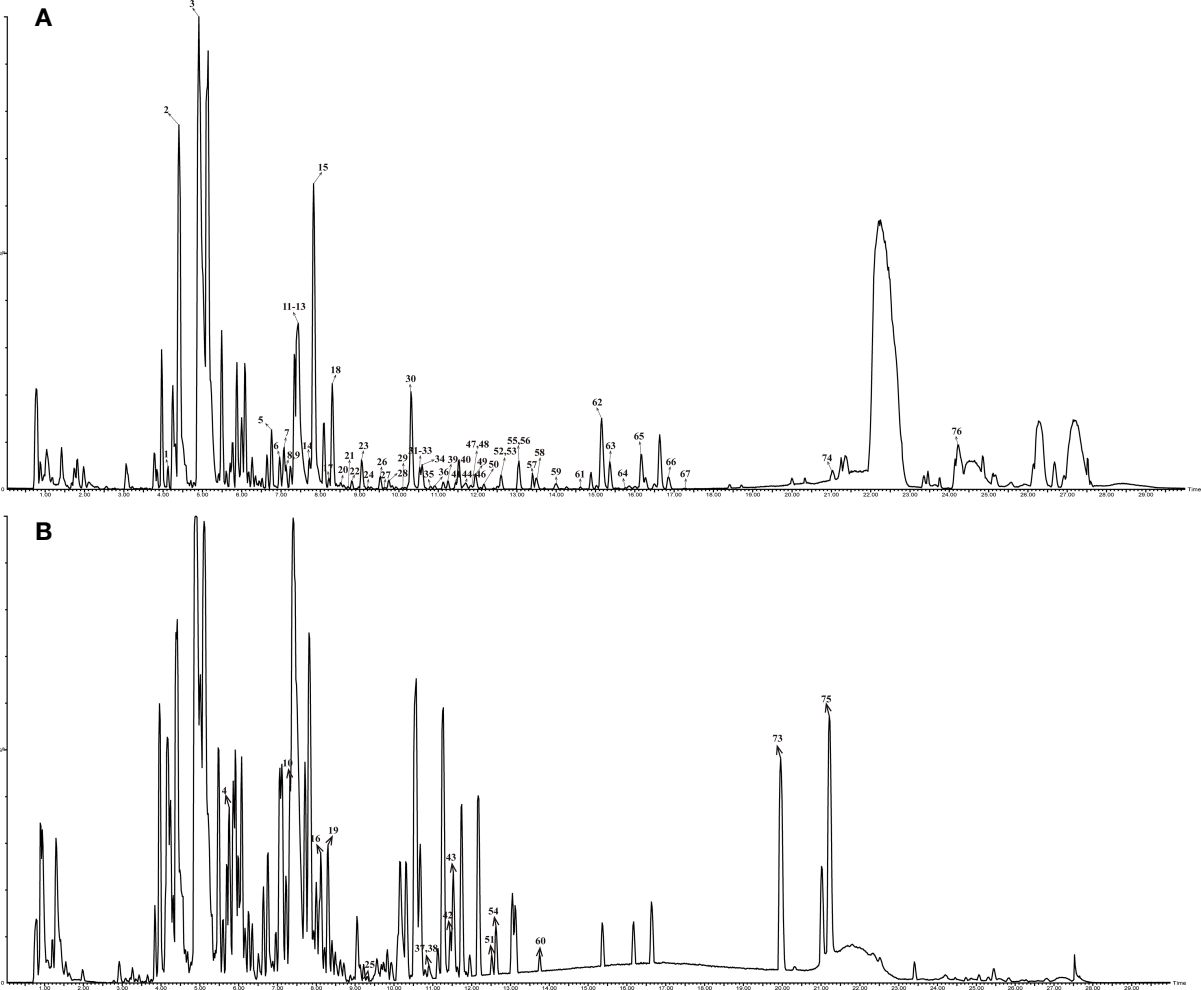
Base peak chromatogram of compounds in Ganshu Nuodan by HPLC-QTOF-MS in different ion modes: **(A)** ESI+; **(B)**ESI-.

The UPLC-QTOF/MS chromatogram of Ganshu Nuodan is shown in **Figure 2**. The data of 76 identified compounds including predicted formula, retention time (tR min), mass error, typical fragment and structural type are shown in **Table S1**.

**Figure 3:**
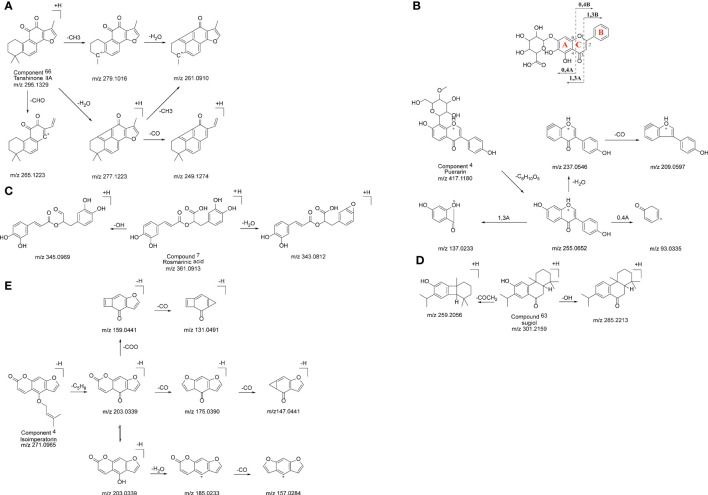
The probable MS fragmentation process of Tanshinone IIA **(A)**, Puerarin **(B)**, Rosmarinic acid **(C)**, sugiol **(D)** and Isoimperatorin **(E)**.

The findings presented in **Table S1** showed that the identified compounds contained 21 quinones, 18 flavonoids, 11 organic acids, 7 terpenoids, 3 coumarins and 16 others. Fragmentation patterns of representative compounds were presented in **Figure 3**.

#### 3.2.1 Identification of quinones

Quinone compounds are a class of aromatic organic compounds containing two double bond six carbon atom ring diketone structure, including naphthoquinones, phenanthrenequinones and anthraquinones. The quinone compounds in *S. miltiorrhiza* are mainly tanshinones which belong to phenanthrenequinones in structure.

Under the positive ion mode, peak 66 displayed a precursor ion [M+H]^+^ at m/z 295.1329 and MS2 fragment ions at m/z 265.1223, 261.0910, and 249.1274. According to the UNIFI software, the elemental formula was suggested to be C_19_H_18_O_3_. Compared with literatures and fragmentation patterns, the compound was speculated to be tanshinone II A, and its possible fragmentation pattern is shown in **Figure 3A**.

#### 3.2.2 Identification of flavonoids

Flavonoids originally referred to a class of compounds derived from 2- phenylchromone skeleton. It is a general term used by a series of compounds connected by two benzene rings through three carbon atoms, that is, a class of compounds with C6-C3-C6 structure. Flavonoids are hyperconjugated systems, mainly composed of aglycones and glucose condensation. As a result of the cleavage of the glycosidic linkage, they can produce the same low m/z fragment ions and stable excimer ion peaks in the positive and negative ion modes. The main MS2 dissociation behavior involved the Retro-Diels-Alder (RDA) fragmentation pathway and consecutive neutral losses of small molecules.

Peak 3 displayed a precursor ion [M+H]^+^ at m/z 417.1180 in the positive ion mode and MS2 fragment ions at m/z 255.0652, 137.0233 and 93.0335. The characteristic ion at m/z 255.0652 was produced by the cleavage of the glycosidic linkage, corresponding to the structure of daidzein. Thus, this compound could be identified as Puerarin. The characteristic ions at m/z 137.0233 and 93.0335 were obtained by the classical fragmentation of flavonoids, and its possible fragmentation mode is shown in **Figure 3B**.

#### 3.2.3 Identification of organic acid

For organic acids, precursor ions showed similar fragmentation patterns and produced small molecules including M-CO_2_, M-CO, and M-H_2_O, which indicated the existence of either carboxyl, carbonyl, or hydroxyl groups. Hence, this strategy showed a great advantage for the identification of the characteristics of organic acids and relevant product ions.

Peak 7 showed a precursor ion [M+H]^+^ at m/z 361.0913under the positive ion mode and MS2 fragment ions at m/z 345.0969 and 343.0812, which were produced by the losses of -OH and -H_2_O group, respectively. Therefore, the compound could be tentatively assigned as Rosmarinic acid by comparing it with the database and previous report. The possible fragmentation pattern was shown in **Figure 3C**.

#### 3.2.4 Identification of terpenoid

Terpenoids are a class of natural compounds and their derivatives are derived from methylglutaric acid with isoprene unit (C5 unit) as the basic molecular skeleton.

Peak 63 gave a precursor ion [M+H]^+^ at m/z 301.2162 in the positive ion mode, and the MS2 spectrum collected fragment ion at m/z 259.1690. By comparing with fragmentation patterns shown in the literature, peak 63 could be tentatively identified as sugiol, and its fragmentation pattern is shown in **Figure 3D**.

#### 3.2.5 Identification of coumarin

Coumarin is a kind of lactone compound formed by intramolecular dehydration cyclization of cis-hydroxycinnamic acid.

A precursor ion [M-H]^-^ of peak 4 at m/z 271.0965 and its MS2 spectrum ions at m/z 131.0491, 147.0441 and 157.0284 were detected by UPLC-QTOF-MS. According to the above information, this compound was identified to be Isoimperatorin. The possible fragmentation pattern was shown in **Figure 3E**.

### 3.3 Results of proteomics

**Figure 4:**
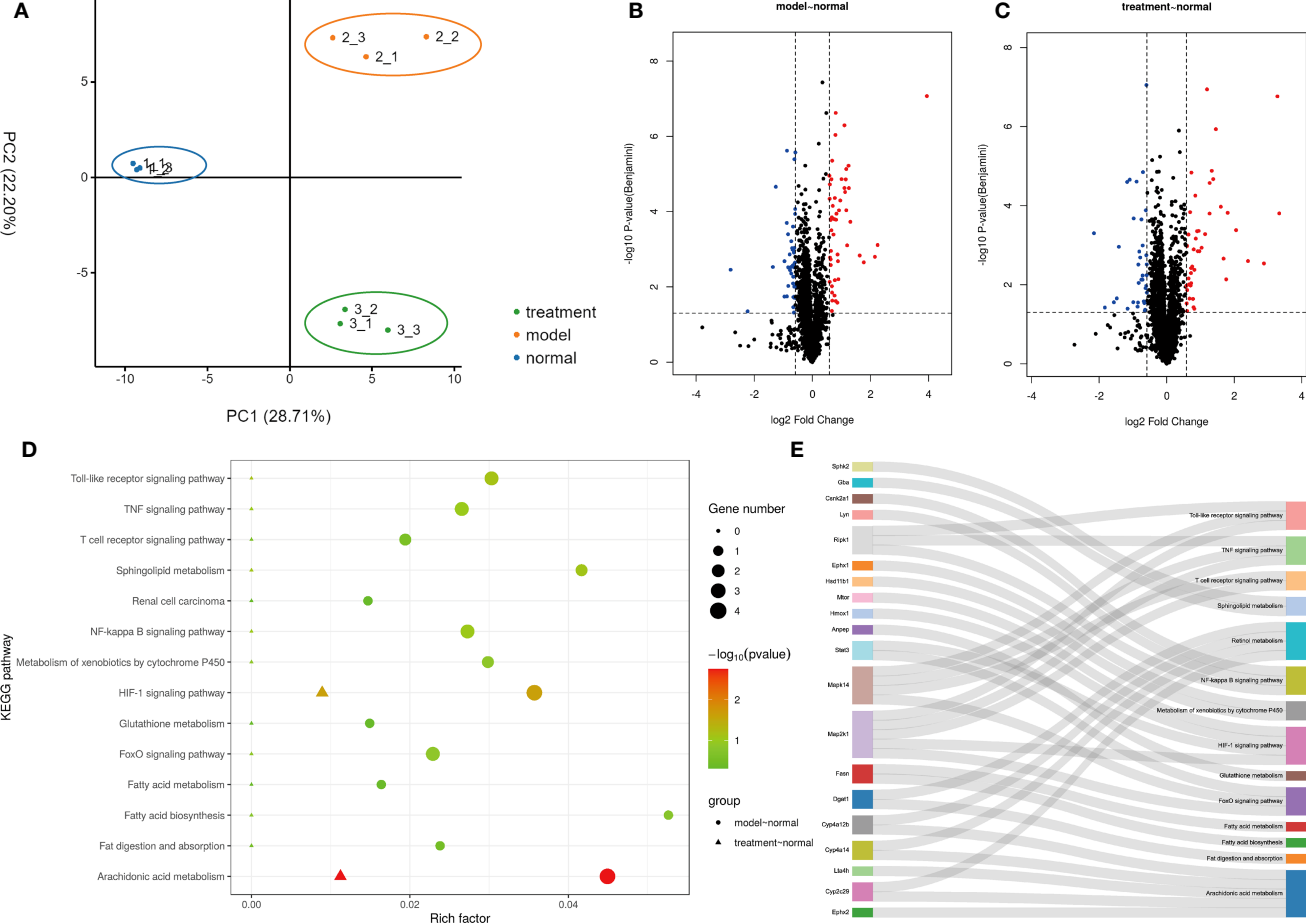
Proteomic Analysis of Ganshu Nuodan. **(A)** The principal component analysis (PCA). **(B, C)** The Volcano plot of Model vs Normal and Treatment vs Normal respectively. **(D)** The KEGG pathway enrichment analysis. **(E)** The sankey dot pathway enrichment analysis.

The results of proteomic analysis are shown in **Figure 4**. A total of 2657 protein groups were identified in all three experimental groups (Normal, Model and Treatment). As shown in **Figure 4A**, there were significant differences in protein expression between the Normal group, the Model group and the Treatment group. Compared with the Normal group, 92 proteins in the Model group changed significantly, of which 51 proteins were up-regulated and 41 proteins were down-regulated (**Figure 4B**). Compared with the Normal group, 79 proteins in the Treatment group changed significantly, of which 44 proteins were up-regulated and 35 proteins were down-regulated (**Figure 4C**). All the above-mentioned differentially expressed proteins were subjected to KEGG pathway enrichment analysis. As shown in **Figure 4D**, there were a total of 14 significantly related pathways. Among them, three pathways are most significantly related, namely Arachidonic acid metabolism, Retinol metabolism and HIF-1 signaling pathway. The genes involved in these pathways are shown in **Figure 4E**. We identified 10 proteins, namely Ephx2, Cyp2c29, Lta4h, Cyp4a14, Cyp4a12b, Map2k1, Stat3, Hmox1, Mtor and Dgat1, that were associated with these three pathways.

### 3.4 Results of network pharmacology

**Figure 5:**
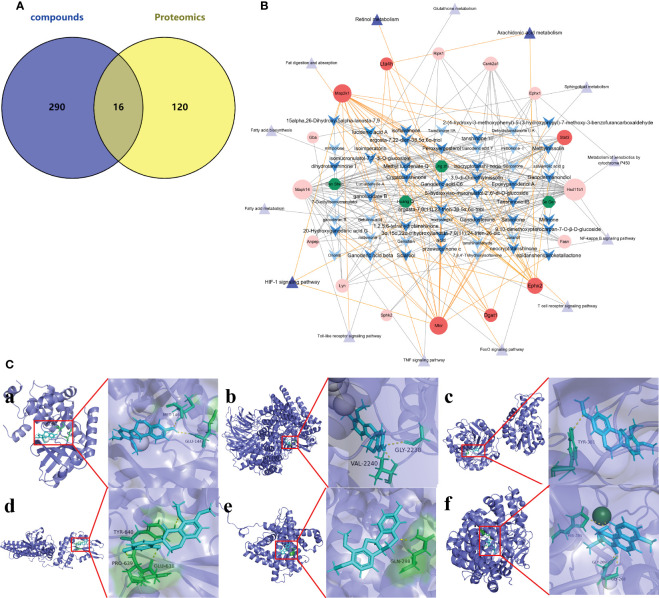
Network Pharmacology of Ganshu Nuodan. **(A)** The venn diagram of Ganshu Nuodan-related component targets and significantly differentially expressed proteins obtained by proteomics. **(B)** The “herbs-compounds-targets-pathways” network of key targets. The green hexagons represent 4 herbs, the blue arrow shapes are chemical components, the red circles are target proteins, and the purple triangles are pathways. The three pathways with darker colors are significantly related pathways obtained by KEGG pathway enrichment analysis, and the targets with darker colors are common targets related to these three pathways. The possible components that regulate these targets are also displayed in darker colors. **(C)** The results of molecular docking. (a) Map2k1 and Methylnissolin, (b) Mtor and Methylnissolin, (c) Ephx2 and 3,9-di-O-methylnissolin, (d) Stat3 and isoflavanone, (e) Dgat1 and Methylnissolin, (f) Lta4h and tanshinone VI.

The 306 action targets of the 76 components identified in Ganshu Nuodan were intersected with 136 significantly differentially expressed proteins screened by proteomics, and a total of 16 common targets were obtained (**Figure 5A**). On this basis, the “component-target-pathway” network was constructed, and the key components in Ganshu Nuodan with improving effect on alcoholic liver disease were obtained (**Figure 5B**), including 5’-hydroxyiso-muronulatol-2’,5’-di-O-glucoside, 3,9-di-O-methylnissolin, isomucronulatol-7,2’-di-O-glucosiole, 15alpha,26-Dihydroxy-5alpha-lanosta-7,9, ergosta-7,22-dien-3β,5α,6α-triol, ergosta-7,9 (8),22-trien-3β,5α,6α-triol, Cryptotanshinone, isocryptotanshi-none, Salviolone, Ganodermanondiol, Ganodosterone, Sclareol, tanshinone VI, Ganoderic acid C6, Ganoderic acid beta, 9,10-dimethoxypterocarpan-3-O-β-D-glucoside, przewaquinone c, isoflavanone, Epoxyganoderiol A, Methylnissolin, Methyl lucidenate Q, 2-(4-hydroxy-3-methoxyphenyl)-5-(3-hydroxypropyl)-7-methoxy-3-benzofurancarboxaldehyde, Tanshinone IIB, ganolucidate B, Peroxyergosterol, 20-Hydroxyganoderic acid G, neocryptotanshinone, dihydrotanshinoneI, 1,2,5,6-tetrahydrotanshinone, Miltrione, Isoimperatorin, epidanshenspiroketallactone, 3α,15α,22α-trihydroxylanosta-7,9 (8),24-trien-26-oic acid, lucidenic acid A. In addition, a total of 6 proteins related to three key pathways were found, including Ephx2 (PDB: 1S8O), Lta1h (PDB: 1GW6), Map2k1 (PDB: 1S9J), Stat3 (PDB: 1BG1), Mtor (PDB: 1AUE) and Dgat1 (PDB: 6VYI).

The 6 key targets were molecular docked with their related compounds, and the binding energy generated by docking is shown in **Table S2**. Among them, the lowest binding energy of each target were shown in **Figure 5C**. Based on the binding energy, 9 key compounds were obtained: Methylnissolin, 2-(4-hydroxy-3-methoxyphenyl)-5-(3-hydroxypropyl)-7-methoxy-7-benzofurancarboxaldehyde, 3,9-di-O-methylnissolin, Salviolone, isoflavanone, Methyl lucidenate Q, 20-Hydroxyganoderic acid G, tanshinone VI and Ganoderic acid C6.

## 4 Discussion

As the leading cause of liver-related diseases worldwide, ALD is caused by heavy drinking. The initial manifestation is the accumulation of lipids, including triglycerides, appearing as a large number of lipid droplets that can be observed by oil red O staining (9). The next step is to develop alcoholic hepatitis, and the most obvious indication is the change of enzymology demonstrated by a significant increase of ALT and AST which are two key biological indicators for evaluating liver function (10, 11). By HE staining, the damaged hepatic lobule structure could be observed, and the hepatic cords were chaotically arranged. Hepatocyte necrosis, fatty degeneration, and vacuolar lipid droplets, ballooning degeneration, and neutrophil infiltration. During this period, liver tissue was enlarged as a result of the swelling of liver cells mainly due to the covalent binding between acetaldehyde, a metabolite of ethanol, and tubulin in the liver which results in the retention of proteins that are normally secreted in the cells (8, 12). In this experiment, Ganshu Nuodan can significantly reduce the liver weight ratio and the levels of ALT and AST in ALD mice. Compared with the model group, the liver sections of the treatment group exhibited fewer lipid droplets, less disruption of hepatic cord arrangement and greatly reduced proportions of vacuolar lipid droplets by oil red O staining and reduced hepatocyte ballooning by HE staining. At present, there are different theories about the pathogenesis of ALD. The main theories are ethanol metabolism, oxidative stress, intestinal dysfunction and lipid accumulation (8, 13–15). In this study, through the construction of a mouse model of acute liver injury, it was found that Ganshu Nuodan mainly functioned by reducing oxidative stress. During the oxidative metabolism of alcohol, a large amount of free radicals will be produced. The accumulation and diffusion of these free radicals will cause oxidative stress, which will damage the structure and function of cells (16). While oxidative stress is considered as a potential mechanism of ALD, it is caused by an imbalance between ROS and antioxidant capacity (17). In addition, ROS can alter the structure and function of proteins by binding to them and ultimately generating host antigens that can induce immune responses. ROS can also lead to lipid peroxidation, and the resulting lipid peroxidation binds to DNA bases, thereby inducing apoptosis and autophagy (18). The oxidative stress in the liver induced by acetaldehyde is related to its high toxicity and reactivity. It causes structural and functional damage to organelles such as mitochondria, which will reduce the production of adenosine triphosphate in the respiratory chain and increase the production of ROS. The excessive ROS can in turn cause oxidative stress to damage the liver (19). ALD severely damages patient’s mitochondria, activate the body’s immune system, induce inflammation, and promote the secretion of ligin, resulting in a large accumulation of collagen in the matrix (14). Therefore, ROS-mediated oxidative stress is the main cause of ALD disease. At the same time, ROS and lipid peroxides can also stimulate liver Kupffer cells to secrete a variety of cytokines (such as tumor necrosis factor TNF-α, interleukins, interferons, chemokines, etc.), and promote liver inflammation, liver cell damage or death (20). MDA is the main final product of lipid peroxidation reaction, and its content can directly reflect the degree of lipid peroxidative damage in the body, while SOD is a superoxide anion scavenger in the body and GSH can remove lipid peroxidation which is harmful to the liver (21). In this experiment, Ganshu Nuodan can significantly reduce the content of MDA in the liver, increase the levels of GSH and SOD in the liver tissue of ALD mice, and demonstrate a protective effect on hepatocyte injury in ALD. Its mechanism may be related to the enhancement of lipid resistance, peroxidation and scavenge oxygen free radicals.

The compounds of Ganshu Nuodan were comprehensively analyzed by UPLC-QTOF/MS and UNIFI combined with the characteristic fragment ions of each compound. A total of 76 compounds were identified, mainly including quinones, flavonoids and organic acids. A total of 18 flavonoids were identified. In addition to the removal of hydroxyl groups, methyl groups, methoxy groups and their glycoside moieties, their characteristic fragmentation mainly occurred in the C ring of the flavonoid nucleus in 1, 4 or 0, 3 modes (22). A total of 21 quinone compounds were identified, and all of them were derived from *S. miltiorrhiza*. The characteristic fragmentation pattern of quinone compounds is to break off the branched chain such as methyl group and remove its main functional group carbonyl group (23). A total of 11 organic acid-like compounds were identified, and it is well known that the most characteristic fragments of this class of compounds are formed by the removal of their carboxyl groups.

Proteomics is a method with the characteristics of “holistic, systematic and comprehensive” that coincides with the holistic view and system view of TCM compounds. Therefore, it is reasonable to use proteomic methods to study the pharmacological mechanism of TCM compounds. A total of 14 main related pathways were obtained by KEGG pathway enrichment analysis of significantly differentially expressed proteins, of which 3 were significantly related, namely Arachidonic acid metabolism, Retinol metabolism and HIF-1 signaling pathway (24–26). Acetaldehyde will damage mitochondria in liver cells, and the generation of free radicals will increase the transcription of COX-2 and activate PLA2, resulting in the production of a large amount of arachidonic acid in the cells and arachidonic acid under the action of COX-2. It will produce prostaglandins and other inflammatory mediators and ROS, leading to aggravation of liver damage (24). Retinol, also known as vitamin A, is an essential dietary precursor for the body’s biosynthesis of retinal and retinoic acid which are mainly found in hepatic stellate cells (27). Retinol metabolism is closely related to the liver, which not only controls the storage and metabolism of this retinol, but also is an important target for the actions of many retinoids (28). Retinol and its metabolites retinal and retinoic acid are also closely related to the occurrence and development of liver diseases. Retinol promotes the deposition of fat in the liver. Retinoic acid in hepatocytes and PAPRγ-mediated CB1R activation promote lipogenesis and inhibit fatty acid oxidation in the liver, thereby aggravating hepatic steatosis. The process by which hepatic stellate cells regulate hepatic inflammatory response is also related to retinol metabolism. Hepatic stellate cells participate in the induction of regulatory T cells, play an antigen-presenting role, activate T cells and cause T cell responses (24). It is currently believed that HIF-1 is a nuclear transcription factor that plays a key role in the regulation of oxygen balance. It can regulate the expression of various hypoxic stress protein genes and participate in various pathophysiological processes (26). Liver tissue hypoxia occurs when drinking alcohol, and meanwhile HIF-1 is activated. Active substances regulated by HIF-1, such as NADPH oxidase, cyclooxygenase, and inducible nitric oxide synthase, are involved in the occurrence of ALD. It has also been shown that loss of HIF-1α exacerbates ALD by inducing intestinal dysbiosis and barrier dysfunction, and that lack of intestinal HIF-1α exacerbates the severity of leaky gut, leading to increased translocation of bacteria and bacterial products leading to ALD.

Through network pharmacology, 34 compounds of Ganshu Nuodan that could potentially improve ALD were obtained, including 9 quinones, 6 flavonoids, 5 organic acids, 4 steroids, 3 terpenes, 3 alcohols, 2 ketones, 1 sterol and 1 coumarin. Quinones came from *S. miltiorrhiza*, named tanshinone, which is the most frequently used herb in clinical treatment of ALD. Tanshinone has strong antioxidant activity, which is beneficial to control the elevation of ALT and AST and inhibit the production of inflammatory mediators by macrophages and monocytes, which can effectively relieve the degeneration and necrosis of liver cells, inhibit the inflammatory response to the liver and promote the regeneration of liver cells. Flavonoids can protect ALD by inhibiting oxidative stress, inflammatory responses, and regulating the hedgehog pathway (29). The flavonoids here all come from *A. membranaceus*, which is an herb for strengthening the body. It has the functions of improving immunity, resisting free radicals and membrane lipid peroxidation, and delaying liver fibrosis. The composition is relatively stable and has been widely used in clinical practice. Long-term intake of alcohol leads to intestinal flora imbalance and increased intestinal permeability, which in turn affects the intestinal barrier function, promotes the migration of intestinal microorganisms and their products into the systemic circulation, induces the accumulation of pro-inflammatory cytokines in the liver, and induces inflammation to accelerate liver damage. On the one hand, organic acids are used as energy substances by the host intestinal wall cells and intestinal probiotics, and on the other hand, by reducing the pH value, they inhibit the growth of pathogenic bacteria such as Escherichia coli and Salmonella and reduce the formation of toxic fermentation products, thereby improving the intestinal microenvironment, protecting the intestinal mucosal barrier function and enhancing the body’s immunity. There are a total of 6 targets related to these 3 pathways, including Lta4h, Ephx2, Dgat1, Map2k1, Stat3 and Mtor. Lta4h is an inflammation-related protein. Ephx2 is a major cytosolic enzyme that is highly expressed in the liver and is associated with liver function, but its role in ALD is largely unexplored. The study found that Ephx2 was significantly increased in the liver of mice after ingesting ethanol, and in Ephx2 knockout mice, Ephx2 deficiency ameliorated ethanol-induced liver damage, inflammation, and steatosis (30). In addition, Ephx2 deficiency was also associated with a marked attenuation of the hepatic endoplasmic reticulum and oxidative stress. Drinking alcohol increases fatty acid flux from the blood to the liver, and Dgat1 acts primarily to esterify exogenous fatty acids to TG (31). In contrast, hepatocyte-specific Dgat1 deletion aggravates alcohol-induced liver injury by increasing lipid accumulation and endoplasmic reticulum stress, reducing LAMP2 protein levels, and impairing autophagy function. The process of ALD is often accompanied by changes in the MAPK signaling pathway, and Map2k1 is one of the main members of the MAPK family, which plays a TCM role in cell growth, differentiation, inflammation and other pathological processes (32). The study found that by inhibiting the expression of MAPK protein, it can effectively alleviate the release of inflammatory factors in ALD mice. After the activation of the MAPK pathway, STAT will be further activated (24). After the activated STAT enters the nucleus, it activates the expression of various genes including malformation stage genes, which can effectively reduce the hepatic inflammatory response and cell damage of alcoholic liver. In contrast, after STAT gene knockout, liver regeneration was reduced and insulin resistance increased. Interestingly, although selective knockout of STAT3 in mouse hepatocytes aggravated the severity of alcoholic fatty liver disease, selective knockout of STAT3 in mouse endothelial cells significantly reduced endothelial and liver damage, suggesting that STAT3 in ALD has different roles in different cells (12). By studying the role of STAT3 in different cells, it was found that STAT3 acts as a pro-inflammatory factor in hepatocytes, which may be related to the release of various cytokines and regulatory proteins by stimulated hepatocytes. STAT3 in gonadal cells plays an anti-inflammatory role as well as a leading role in alcoholic liver injury. Finally, molecular docking confirmed that these targets did have good docking with their related chemical components.

## 5 Conclusion

Via UPLC-QTOF/MS and proteomics, this study confirmed that Ganshu Nuodan may mediate Arachidonic acid metabolism, Retinol metabolism and HIF-1 signaling pathway in animal models, which may play a protective role in ALD by regulating Ephx2, Lta4h, Map2k1, Stat3, Mtor and Dgat1, providing evidence for subsequent studies. However, this study was limited to animal models and did not involve trials to confirm the hepatoprotective activity of compounds.

## Data availability statement

The mass spectrometry proteomics data have been deposited to the ProteomeXchange Consortium (http://proteomecentral.proteomexchange.org) via the iProX partner repository with the dataset identifier PXD045221. Tables of compounds identification and the binding energy information are included in the article/Supplementary Material. Further inquiries can be directed to the corresponding authors.

## Ethics statement

The animal studies were approved by Nanjing Agricultural University. The studies were conducted in accordance with the local legislation and institutional requirements. Written informed consent was obtained from the owners for the participation of their animals in this study.

## Author contributions

XY, LW and XC: data curation and writing-original draft preparation; CD, YW and MD: methodology and conceptualization. JZ, ZL, BZ, ZJ, RY: data validation and curation. RY, YL, YW, KW: writing-reviewing, visualization and editing. SQ, CD, GZ: conceptualization, funding acquisition and project administration.
