# Supplementary material for: Proteomics and network pharmacology of Ganshu Nuodan capsules in the prevention of alcoholic liver disease
Source: Front Endocrinol (Lausanne). 2023 Sep 18;14:1229777. doi: 10.3389/fendo.2023.1229777 (PMC10547213; doi:10.3389/fendo.2023.1229777)
Supplement: Supplementary file 1 [file Table_1.docx]

Supplementary Material

Study of Ganshu Nuodan in protecting against alcohol liver disease using proteomics and network pharmacology

# Supplementary Table 1. Compounds identified in Ganshu Nuodan using HPLC-QTOF-MS

| No | t_R_  min | Predicted formula | | Neutral  mass | Observed  m/z | mass error  （mDa） | MS/MS | Adduct | Component name | Herb | Structure |
| --- | --- | --- | --- | --- | --- | --- | --- | --- | --- | --- | --- |
| 1 | 4.11 | | C_20_H_18_O_10_ | 418.0900 | 419.0971 | -0.1 | 299.0538 [C_16_H_11_O_6_]^+^, 137.0230[C_7_H_5_O_3_]^+^ | +H | Bifendate | LZ | others |
| 2 | 4.41 | | C_17_H_12_O_6_ | 312.0634 | 313.0710 | 0.3 | 133.0281 [C_8_H_5_O_2_]^+^, 110.0252 [C_6_H_6_O_2_]^+^ | +H | Tournefolic acid A | HQ | organic acid |
| 3 | 4.92 | | C_21_H_20_O_9_ | 416.1180 | 417.1180 | 0.2 | 255.0652 [C_15_H_11_O_4_]^+^, 137.0233 [C_7_H_5_O_3_]^+^, 93.0335 [C_6_H_5_O]^+^ | +H, +Na | Puerarin | GG | flavonoid |
| 4 | 5.7 | | C_16_H_14_O_4_ | 270.0892 | 269.0812 | -0.7 | 150.0307 [C_8_H_6_O_3_]^-^, 243.0653 [C_14_H_11_O_4_]^-^, 177.0549 [C_10_H_9_O_3_]^-^ | -H | Isoimperatorin | DS | coumarin |
| 5 | 6.77 | | C_22_H_22_O_9_ | 430.1264 | 431.1336 | -0.1 | 139.0388 [C_7_H_7_O_3_]^+^, 267.0648 [C_16_H_11_O_4_]^+^,239.0692 [C_15_H_11_O_3_]^+^ | +H, +Na | Ononin | DS | flavonoid |
| 6 | 6.97 | | C_15_H_10_O_5_ | 270.0528 | 271.0602 | 0.1 | 253.0492 [C_15_H_9_O_4_]^+^, 137.023 [C_7_H_5_O_3_]^+^ | +H | 7,8,4'-Trihydroxyisoflavone | LZ | flavonoid |
| 7 | 7.08 | | C_18_H_16_O_8_ | 360.0845 | 361.0913 | -0.5 | 163.0391 [C_9_H_7_O_3_]^+^, 145.0281 [C_9_H_5_O_2_]^+^ | +H, +Na | Rosmarinic acid | DS | organic acid |
| 8 | 7.12 | | C_11_H_10_O_4_ | 206.0579 | 207.0650 | -0.2 | 153.0533 [C_8_H_9_O_3_]^+^, 163.0391[C_9_H_7_O_3_]^+^ | +H | Scoparone | DS | coumarin |
| 9 | 7.14 | | C_18_H_12_O_7_ | 340.0583 | 341.0656 | 0.1 | 295.0599 [C_17_H_11_O_5_]^+^, 279.0648 [C_17_H_11_O_4_]^+^ | +H | salvianolic acid | DS | organic acid |
| 10 | 7.34 | | C_29_H_38_O_16_ | 642.216 | 641.2083 | -0.4 | 193.0503 [C_10_H_9_O_4_]^-^, 317.1014 [C_17_H_17_O_6_]^-^ | -H | 5'-hydroxyiso-muronulatol-2',5'-di-O-glucoside | LZ | flavonoid |
| 11 | 7.4 | | C_18_H_16_O_5_ | 312.0998 | 313.1064 | -0.7 | 249.0545 [C_16_H_9_O_3_]^+^, 111.0437 [C_6_H_7_O_2_]^+^, 137.023 [C_7_H_5_O_3_]^+^ | +H | przewaquinone f | DS | quinone |
| 12 | 7.44 | | C_27_H_22_O_12_ | 538.1111 | 539.1181 | -0.3 | 323.0551 [C_18_H_11_O_6_]^+^，295.0604 [C_17_H_11_O_5_]^+^ | +H | salvianolic acid j | DS | organic acid |
| 13 | 7.54 | | C_18_H_18_O_5_ | 314.1154 | 315.1224 | -0.3 | 283.0594 [C_16_H_11_O_5_]^+^, 283.0602[C_16_H_11_O_5_]^+^, 253.0494[C_15_H_9_O_4_]^+^ | +H, +Na | 3,9-di-O-methylnissolin | LZ | flavonoid |
| 14 | 7.71 | | C_21_H_18_O_11_ | 446.0849 | 447.0933 | 1.1 | 269.0425 [C_15_H_9_O_5_]^+^，146.0348 [C_9_H_6_O_2_]^+^ | +H | Baicalin | LZ | flavonoid |
| 15 | 7.84 | | C_15_H_10_O_4_ | 254.0579 | 255.0652 | 0.1 | 137.0231 [C_7_H_5_O_3_]^+^, 119.0485 [C_8_H_7_O]^+^, 109.0278 [C_6_H_5_O_2_]^+^ | +H, +Na | Daidzein | LZ | flavonoid |
| 16 | 8.17 | | C_29_H_38_O_15_ | 626.2211 | 625.2121 | -1.7 | 267.0655 [C_16_H_11_O_4_]^-^, 299.0911 [C_17_H_15_O_5_]^-^, 282.0526 [C_16_H_10_O_5_]^-^ | -H, +HCOO | isomucronulatol-7,2'-di-O-glucosiole | DS | flavonoid |
| 17 | 8.21 | | C_23_H_26_O_10_ | 462.1526 | 485.1422 | 0.4 | 299.0911 [C_17_H_15_O_5_]^+^, 284.0679 [C_16_H_12_O_5_]^+^ | +Na | 9,10-dimethoxypterocarpan-3-O-β-D-glucoside | LZ | flavonoid |
| 18 | 8.31 | | C_16_H_12_O_5_ | 284.0685 | 285.076 | 0.3 | 253.0495 [C_15_H_9_O_4_]^+^, 269.0442 [C_15_H_9_O_5_]^+^, 133.0279 [C_8_H_5_O_2_]^+^ | +H, +Na | 3'-Methoxydaidzein | LZ | flavonoid |
| 19 | 8.31 | | C_16_H_12_O_5_ | 284.0685 | 283.0611 | -0.1 | 268.0374 [C_15_H_8_O_5_]^-^, 135.0082 [C_7_H_3_O_3_]^-^, 132.0213 [C_8_H_4_O_2_]^-^ | -H | Calycosin | LZ | flavonoid |
| 20 | 8.54 | | C_17_H_14_O_6_ | 314.079 | 315.0863 | 0 | 300.0628 [C_16_H_12_O_6_]^+^, 167.0339 [C_8_H_7_O_4_]^+^ | +H, +Na | Jaranol | DS | flavonoid |
| 21 | 8.76 | | C_18_H_16_O_5_ | 312.0998 | 313.1068 | -0.2 | 221.0956 [C_16_H_13_O]^+^, 285.0755[C_16_H_13_O_5_]^+^, 267.1012[C_17_H_15_O_3_]^+^ | +H, +Na | (6S,7R)-6,7-dihydroxy-1,6-dimethyl-8,9-dihydro-7H-naphtho[8,7-g]benzofuran-10,11-dione | LZ | quinone |
| 22 | 8.76 | | C_17_H_14_O_3_ | 266.0943 | 267.1012 | -0.3 | 202.0770 [C_16_H_10_]^+^, 221.0956 [C_16_H_13_O]^+^, 249.0907 [C_17_H_13_O_2_]^+^ | +H | dihydrotanshinlactone | DS | coumarin |
| 23 | 9.06 | | C_15_H_10_O_5_ | 270.0528 | 271.0603 | 0.2 | 253.0490 [C_15_H_9_O_4_]^+^, 145.0278 [C_9_H_5_O_2_]^+^, 119.0489 [C_8_H_7_O]^+^ | +H | Genistein | DS | flavonoid |
| 24 | 9.27 | | C_18_H_16_O_4_ | 296.1049 | 297.1118 | -0.3 | 255.0652 [C_15_H_11_O_4_]^+^, 269.0806 [C_16_H_13_O_4_]^+^ | +H, +Na | przewaquinone c | DS | quinone |
| 25 | 9.36 | | C_17_H_16_O_6_ | 316.0947 | 315.0861 | -1.3 | 284.0311 [C_15_H_8_O_6_]^-^, 119.0496 [C_8_H_7_O]^-^, 137.0236 [C_7_H_5_O_3_]^-^ | -H | isoflavanone | DS | flavonoid |
| 26 | 9.44 | | C_18_H_16_O_4_ | 296.1049 | 297.1116 | -0.5 | 249.09 [C_17_H_13_O_2_]^+^, 202.0775 [C_16_H_10_]^+^ | +H, +Na | tanshinone Ⅵ | DS | quinone |
| 27 | 9.68 | | C_30_H_46_O_2_ | 438.3498 | 439.3563 | -0.7 | 421.3455 [C_30_H_45_O]^+^, 145.10045 [C_11_H_13_]^+^ | +H | Lucialdehyde A | DS | terpenoid |
| 28 | 9.75 | | C_19_H_20_O_4_ | 312.1362 | 313.1437 | 0.3 | 249.0908 [C_17_H_13_O_2_]^+^, 225.0530 [C_14_H_9_O_3_]^+^ | +H | miltionone Ⅱ | DS | quinone |
| 29 | 10.08 | | C_19_H_20_O_4_ | 312.1362 | 313.1433 | -0.2 | 251.1062 [C_17_H_15_O_2_]^+^, 225.0539 [C_14_H_9_O_3_]^+^ | +H, +Na | miltionone Ⅰ | DS | quinone |
| 30 | 10.32 | | C_16_H_12_O_4_ | 268.0736 | 269.0812 | 0.3 | 254.0564 [C_15_H_10_O_4_]^+^, 118.0490 [C_8_H_6_O]^+^, 137.0229 [C_7_H_5_O_3_]^+^, | +H, +Na | Formononetin | GG  HQ | flavonoid |
| 31 | 10.54 | | C_30_H_48_O_4_ | 472.3553 | 473.3623 | -0.2 | 297.2212 [C_21_H_29_O]^+^, 437.3412 [C_30_H_45_O_2_]^+^, 455.3516 [C_30_H_47_O_3_]^+^ | +H | Epoxyganoderiol A | DS | terpenoid |
| 32 | 10.54 | | C_30_H_46_O_3_ | 454.3447 | 455.3518 | -0.2 | 143.1064 [C_8_H_15_O_2_]^+^, 437.3412 [C_30_H_45_O_2_]^+^, 419.3304 [C_30_H_43_O]^+^ | +H | Ganoderic acid Y | DS | organic acid |
| 33 | 10.56 | | C_18_H_12_O_4_ | 292.0736 | 293.0807 | -0.2 | 205.0646 [C_15_H_9_O]^+^, 219.0789 [C_16_H_11_O]^+^ | +H | Przewaquinone B | DS | quinone |
| 34 | 10.6 | | C_17_H_16_O_5_ | 300.0998 | 301.1073 | 0.2 | 152.0462 [C_8_H_8_O_3_]^+^, 167.0704 [C_9_H_11_O_3_]^+^, 134.0359 [C_8_H_6_O_2_]^+^ | +H, +Na | Methylnissolin | DS | flavonoid |
| 35 | 10.81 | | C_17_H_18_O_5_ | 302.1154 | 303.1224 | -0.3 | 149.0590 [C_9_H_9_O_2_]^+^, 106.0404 [C_7_H_6_O]^+^ | +H | Isomucrnulatol | DS | flavonoid |
| 36 | 10.91 | | C_18_H_14_O_4_ | 294.0892 | 295.0965 | 0 | 169.0640 [C_12_H_9_O]^+^, 221.0944 [C_16_H_13_O]^+^ | +H, +Na | 3-beta-Hydroxymethyllenetanshiquinone | LZ | quinone |
| 37 | 10.91 | | C_27_H_38_O_6_ | 458.2668 | 457.2582 | -1.4 | 253.1220 [C_17_H_17_O_2_]^-^, 267.1009 [C_17_H_15_O_3_]^-^, 211.1332 [C_12_H_19_O_3_]^-^ | -H | lucidenic acid A | DS | organic acid |
| 38 | 10.94 | | C_30_H_42_O_8_ | 530.2880 | 529.2798 | -0.8 | 357.1693 [C_21_H_25_O_5_]^-^, 511.2688 [C_30_H_39_O_7­_]^-^, 457.2586 [C_27_H_37_O_6_]^-^ | -H | Ganoderic acid C6 | DS | organic acid |
| 39 | 11.2 | | C_28_H_42_O_6_ | 474.2981 | 497.289 | 1.6 | 437.3412 [C_30_H_45_O_2_]^+^, 256.1105 [C_16_H_16_O_3_]^+^ | +Na | Methyl lucidenate Q | DS | terpenoid |
| 40 | 11.24 | | C_18_H_20_O_3_ | 284.1412 | 285.1483 | -0.2 | 142.0760 [C_11_H_10_]^+^, 85.0282 [C_4_H_5_O_2_]^+^ | +H | epidanshenspiroketallactone | DS | others |
| 41 | 11.46 | | C_18_H_20_O_5_ | 316.1311 | 339.1221 | 1.9 | 177.0902 [C_11_H_13_O_2_]^+^, 143.1064 [C_8_H_15_O_2_]^+^, 125.0958 [C_8_H_13_O]^+^ | +Na | 7-O-ethylisomucronulatol | LZ | flavonoid |
| 42 | 11.46 | | C_20_H_20_O_6_ | 356.126 | 355.1176 | -1.1 | 239.0334 [C_14_H_7_O_4_]^-^, 295.0600 [C_17­_H­_11_O_5_]^-^, 323.0912 [C_19_H_15_O_5_]^-^ | -H | 2-(4-hydroxy-3-methoxyphenyl)-5-(3-hydroxypropyl)-7-methoxy-3-benzofurancarboxaldehyde | DS | others |
| 43 | 11.52 | | C_30_H_44_O_6_ | 500.3138 | 499.3054 | -1.1 | 437.3044 [C_29_H_41_O_3_]^-^, 285.1842 [C_19_H_25_O_2_]^-^, 481.2943 [C_30_H_41_O_5_]^-^ | -H | Ganoderic acid beta | DS | organic acid |
| 44 | 11.61 | | C_17_H_12_O_4_ | 280.0736 | 281.0805 | -0.3 | 224.0815 [C_15_H_12_O_2_]^+^, 225.0537 [C_14_H_9_O_3_]^+^ | +H, +Na | Nortanshinone | DS | quinone |
| 45 | 11.64 | | C_30_H_44_O_9_ | 548.2985 | 571.2895 | 1.8 | 407.2786 [C_24_H_39_O_5_]^+^, 321.1850 [C_22_H_25_O_2_]^+^ | +Na | 20-Hydroxyganoderic acid G | LZ | organic acid |
| 46 | 11.69 | | C_18_H_18_O_3_ | 282.1256 | 283.1324 | -0.5 | 265.1220 [C_18_H_17_O_2_]^+^, 185.0977 [C_13_H_13_O]^+^, 208.0868 [C_15_H_12_O]^+^ | +H | danshenspiroketallactone | DS | quinone |
| 47 | 11.84 | | C_19_H_18_O_4_ | 310.1205 | 311.128 | 0.3 | 293.1165 [C_19_H_17_O_3_]^+^, 141.0696 [C_11_H_9_]^+^ | +H, +Na | 3α-hydroxytanshinoneⅡa | LZ | quinone |
| 48 | 11.87 | | C_19_H_16_O_3_ | 292.1099 | 293.1168 | -0.4 | 219.0794 [C_16_H_11_O]^+^, 235.1110 [C_17_H_15_O]^+^ | +H | Dehydrotanshinone II A | DS | quinone |
| 49 | 11.95 | | C_19_H_18_O_4_ | 310.1205 | 311.1281 | 0.3 | 185.0955 [C_13_H_13_O]^+^, 252.1141 [C_17_H_16_O_2_]^+^, 237.0904 [C_16_H_13_O_2_]^+^ | +H | TanshinoneIIB | DS | quinone |
| 50 | 12.16 | | C_30_H_46_O_3_ | 454.3447 | 455.3509 | -1 | 297.2206 [C_21_H_29_O]^+^, 437.3409 [C­_30_H_45_O_2_]^+^, 419.3302 [C_30_H_43_O]^+^ | +H | 15alpha,26-Dihydroxy-5alpha-lanosta-7,9(11),24-triene-3-one | LZ | others |
| 51 | 12.46 | | C_30_H_46_O_5_ | 486.3345 | 485.3265 | -0.8 | 277.2161 [C_18_H_29_O_2_]^-^, 171.10208 [C_9_H_15_O_3_]^-^, 221.1540 [C_14_H_21_O_2_]^-^ | -H, +HCOO | 3α,15α,22α-trihydroxylanosta-7,9(11),24-trien-26-oic acid | LZ | organic acid |
| 52 | 12.6 | | C_19_H_16_O_4_ | 308.1049 | 309.1121 | -0.1 | 265.0844 [C_17_H_13_O_3_]^+^, 279.1008 [C_18_H_15_O_3_]^+^ | +H | tanshinaldehyde | DS | quinone |
| 53 | 12.88 | | C_28_H_46_O_3_ | 430.3447 | 453.3352 | 1.3 | 201.1632 [C_15_H_21_]^+^, 205.1582 [C_14_H_21_O]^+^, 187,1475 [C_14_H_19_]^+^ | +Na | ergosta-7,22-dien-3β,5α,6α-triol | DS | others |
| 54 | 12.88 | | C_29_H_46_O_3_ | 442.3447 | 487.3424 | -0.5 | 269.1164 [C_17_H_17_O_3_]^-^, 237.0917 [C_16_H_13_O_2_]^-^, 221.0617 [C_15_H_9­_O_2_]^-^ | +HCOO | ergosta-7,9(11),22-trien-3β,5α,6α-triol | DS | others |
| 55 | 13.05 | | C_19_H_22_O_4_ | 314.1518 | 337.1407 | -0.3 | 269.1529 [C_18_H_21_O_2_]^+^, 297.1481 [C_19_H_21_O_3_]^+^ | +Na, +H | neocryptotanshinone | DS | quinone |
| 56 | 13.05 | | C_19_H_20_O_3_ | 296.1412 | 297.1484 | -0.1 | 143.0835 [C_11_H_11_]^+^, 269.1534 [C_18_H_21_O_2_]^+^, 141.0693 [C_11_H_9_]^+^ | +H | Cryptotanshinone | LZ | quinone |
| 57 | 13.34 | | C_19_H_22_O_3_ | 298.1569 | 299.1637 | -0.4 | 269.1532 [C_18_H_21_O_2_]^+^, 252.113 [C_17_H_16_O_2_]^+^, 239.1056 [C_16_H_15_O_2_]^+^ | +H, +Na | deoxyneocryptotanshinone | DS | quinone |
| 58 | 13.5 | | C_18_H_14_O_3_ | 278.0943 | 279.1014 | -0.2 | 261.0907 [C_18_H_13_O_2_]^+^, 221.0583 [C_15_H_9_O_2_]^+^ | +H, +Na | dihydrotanshinoneⅠ | DS | quinone |
| 59 | 14 | | C_18_H_16_O_3_ | 280.1099 | 281.117 | -0.2 | 263.1059 [C_18_H_15_O_2_]^+^, 127.0539 [C_10_H_7_]^+^ | +H, +Na | 1,2,5,6-tetrahydrotanshinone | DS | quinone |
| 60 | 14.06 | | C_20_H_26_O_2_ | 298.1933 | 297.1848 | -1.2 | 282.1610 [C_19_H_22_O_2_]^-^ | -H | microstegiol | DS | others |
| 61 | 14.47 | | C_19_H_24_O_3_ | 300.1725 | 301.1792 | -0.6 | 241.1216 [C_16_H_17_O_2_]^+^, 271.1689 [C_18_H_23_O_2_]^+^,  256.1451 [C_17_H_20_O_2_]^+^ | +H | miltipolone | DS | others |
| 62 | 15.16 | | C_19_H_20_O_3_ | 296.1412 | 297.1486 | 0.1 | 279.1379 [C_19_H_19_O_2_]^+^, 254.0934 [C_16_H_14_O_3_]^+^ | +H, +Na | isocryptotanshi-none | DS | quinone |
| 63 | 15.31 | | C_20_H_28_O_2_ | 300.2089 | 301.2159 | -0.3 | 259.1690 [C_17_H_23_O_2_]^+^, 213.1267 [C_15_H_17_O]^+^, 95.08518 [C_7_H_11_]^+^ | +H | sugiol | DS | terpenoid |
| 64 | 15.68 | | C_23_H_26_O_6_ | 398.1729 | 421.1637 | 1.5 | 319.0961 [C_20_H_15_O_4_]^+^, 281.0462 [C_16_H_9_O_5_]^+^_­_, 337.1054 [C_20_H_17_O_5_]^+^ | +Na | przewalskin a | DS | terpenoid |
| 65 | 16.19 | | C_18_H_20_O_2_ | 268.1463 | 269.1535 | -0.1 | 239.1062 [C_16_H_15_O_2_]^+^, 141.0694 [C_11_H_9_]^+^, 254.1295 [C_17_H_18_O_2_]^+^ | +H | Salviolone | DS | others |
| 66 | 16.86 | | C_19_H_18_O_3_ | 294.1256 | 295.1329 | -0.2 | 265.1223 [C_18_H_17_O_2_]^+^, 261.0910 [C_18_H_13_O_2_]^+^, 249.1274 [C_18_H_16_O]^+^ | +H, +Na | Tanshinone IIA | DS | quinone |
| 67 | 17.37 | | C_19_H_22_O_2_ | 282.162 | 283.1694 | 0.1 | 269.0805 [C_16_H_13_O_4_]^+^, 239.1061 [C_16_H_15_O_2_]^+^ | +H, +Na | Miltrione | DS | others |
| 68 | 18.1 | | C_31_H_48_O_6_ | 516.3451 | 515.3366 | -1.2 | 169.0861 [C_9_H_13_O_3_]^-^, 453.2995 [C_29_H_41_O_4_]^-^, 125.0967 [C_8_H_13_O]^-^ | -H | ganolucidate B | DS | terpenoid |
| 69 | 18.37 | | C_30_H_48_O_3_ | 456.3604 | 455.3518 | -1.3 | 255.2328 [C_16_H_31_O_2_]^-^, 277.2165 [C_18_H_29_O_2_]^-^, 299.2002 [C_20_H_27­_O_2_]^-^ | -H, +HCOO | Ganodermanondiol | DS | terpenoid |
| 70 | 18.73 | | C_20_H_30_O | 286.2297 | 331.2276 | -0.3 | 135.0808 [C_9_H_11_O]^-^, 219.1750 [C_15_H_23_O]^-^, 161.0965 [C_11_H_13_O]^-^ | +HCOO | Ferruginol | DS | others |
| 71 | 18.84 | | C_30_H_48_O_3_ | 456.3604 | 455.3525 | -0.5 | 277.2166 [C_18_H_29_O_2_]^-^, 325.2523 [C_23_H_33_O]^-^, 409.2737 [C_27_H_37­_O_3_]^-^ | -H, +HCOO | Betulinic acid | LZ | organic acid |
| 72 | 19.94 | | C_30_H_46_O_3_ | 454.3447 | 453.3376 | 0.2 | 125.0968 [C_8_H_13_O]^-^, 261.2222 [C_18_H_29_O]^-^, 275.2003 [C_18_H_27_O]^-^ | -H, +HCOO | ganoderan B | DS | others |
| 73 | 20.12 | | C_28_H_44_O_4_ | 444.3240 | 489.3214 | -0.8 | 239.1429 [C_17_H_19_O]^-^, 369.2780 [C_26_H_37_O_2_]^-^, 283.1321 [C_18_H_19_O_3_]^-^ | +HCOO | Peroxyergosterol | DS | others |
| 74 | 21.13 | | C_28_H_40_O_2_ | 408.3028 | 409.309 | -1.1 | 283.1693 [C_19_H_23_O_2_]^+^ | +H | Ganodosterone | DS | others |
| 75 | 21.46 | | C_20_H_36_O_2_ | 308.2715 | 307.2648 | 0.5 | 125.0971 [C_8_H_13_O]^-^ | -H | Sclareol | DS | others |
| 76 | 24.32 | | C_28_H_40_O | 392.3079 | 393.3155 | 0.3 | 268.1823 [C_19_H_24_O]^+^, 253.1585 [C_18_H_21_O]^+^, 224.1555 [C_17_H_20_]^+^ | +H | Ergosta-4,6,8(14),22-tetraen-3-one | DS | others |

* LZ represents G. lucidum, GG represents P. montana, DS represents S. miltiorrhiza, HQ represents A. membranaceus

**Supplementary** **Table 2.** The binding energy of targets and compounds

| **targets** | **compounds** | **binding energy**  **(kcal/mol)** |
| --- | --- | --- |
| Map2k1 | Methylnissolin | -8.904 |
| Map2k1 | 2-(4-hydroxy-3-methoxyphenyl)-5-(3-hydroxypropyl)-7-methoxy-7-benzofurancarboxaldehyde | -8.331 |
| Map2k1 | 9,10-dimethoxypterocarpan-3-O-β-D-glucoside | -7.57 |
| Map2k1 | isoflavanone | -6.982 |
| Map2k1 | TanshinoneIIB | -6.971 |
| Map2k1 | przewaquinone c | -6.599 |
| Map2k1 | 3,9-di-O-methylnissolin | -6.034 |
| Map2k1 | Methyl lucidenate Q | -4.752 |
| Map2k1 | ergosta-7,9(11),22-trien-3β,5α,6α-triol | -4.283 |
| Map2k1 | Epoxyganoderiol A | -4.138 |
| Mtor | 2-(4-hydroxy-3-methoxyphenyl)-5-(3-hydroxypropyl)-7-methoxy-6-benzofurancarboxaldehyde | -8.773 |
| Mtor | Methylnissolin | -8.289 |
| Mtor | epidanshenspiroketallactone | -6.96 |
| Mtor | 3,9-di-O-methylnissolin | -6.885 |
| Mtor | 20-Hydroxyganoderic acid G | -6.585 |
| Mtor | ganolucidate B | -6.546 |
| Mtor | Isoimperatorin | -6.536 |
| Mtor | TanshinoneIIB | -6.411 |
| Mtor | Epoxyganoderiol A | -6.016 |
| Mtor | Ganoderic acid beta | -5.623 |
| Mtor | Methyl lucidenate Q | -5.363 |
| Mtor | Peroxyergosterol | -5.331 |
| Mtor | 15alpha,26-Dihydroxy-5alpha-lanosta-7,9 | -5.187 |
| Mtor | ergosta-7,22-dien-3β,5α,6α-triol | -4.934 |
| Mtor | ergosta-7,9(11),22-trien-3β,5α,6α-triol | -4.869 |
| Mtor | Ganoderic acid C6 | -4.181 |
| Ephx2 | 3,9-di-O-methylnissolin | -7.639 |
| Ephx2 | Salviolone | -7.198 |
| Ephx2 | Cryptotanshinone | -7.084 |
| Ephx2 | isocryptotanshi-none | -6.584 |
| Stat3 | isoflavanone | -5.139 |
| Stat3 | Methyl lucidenate Q | -4.453 |
| Stat3 | 1,2,5,6-tetrahydrotanshinone | -4.285 |
| Stat3 | Miltrione | -4.244 |
| Stat3 | 20-Hydroxyganoderic acid G | -4.227 |
| Stat3 | dihydrotanshinoneⅠ | -4.123 |
| Stat3 | Cryptotanshinone | -3.87 |
| Stat3 | 3,9-di-O-methylnissolin | -3.794 |
| Stat3 | neocryptotanshinone | -3.476 |
| Dgat1 | Methylnissolin | -5.785 |
| Dgat1 | 20-Hydroxyganoderic acid G | -5.001 |
| Dgat1 | ganolucidate B | -4.163 |
| Dgat1 | lucidenic acid A | -4.078 |
| Lta4h | Ganoderic acid C6 | -6.427 |
| Lta4h | tanshinone Ⅵ | -5.989 |
| Lta4h | Sclareol | -4.091 |
